# Intertrip consistency in hunting behavior improves foraging success and efficiency in a marine top predator

**DOI:** 10.1002/ece3.7337

**Published:** 2021-03-13

**Authors:** Cassie N. Speakman, Sebastian T. Lloyd, Elodie C. M. Camprasse, Andrew J. Hoskins, Mark A. Hindell, Daniel P. Costa, John P. Y. Arnould

**Affiliations:** ^1^ School of Life and Environmental Sciences Deakin University Burwood Vic. Australia; ^2^ CSIRO Health and Biosecurity Townsville Qld Australia; ^3^ Institute for Marine and Antarctic Studies University of Tasmania Hobart Tas Australia; ^4^ Ecology and Evolutionary Biology Department University of California Santa Cruz Santa Cruz CA USA

**Keywords:** central‐place foraging, foraging behavior, intraindividual variation, marine predator, repeatability, specialization

## Abstract

Substantial variation in foraging strategies can exist within populations, even those typically regarded as generalists. Specializations arise from the consistent exploitation of a narrow behavioral, spatial or dietary niche over time, which may reduce intraspecific competition and influence adaptability to environmental change. However, few studies have investigated whether behavioral consistency confers benefits at the individual and/or population level. While still recovering from commercial sealing overexploitation, Australian fur seals (AUFS; *Arctocephalus pusillus doriferus*) represent the largest marine predator biomass in south‐eastern Australia. During lactation, female AUFS adopt a central‐place foraging strategy and are, thus, vulnerable to changes in prey availability. The present study investigated the population‐level repeatability and individual consistency in foraging behavior of 34 lactating female AUFS at a south‐east Australian breeding colony between 2006 and 2019. Additionally, the influence of individual‐level behavioral consistency on indices of foraging success and efficiency during benthic diving was determined. Low to moderate population‐level repeatability was observed across foraging behaviors, with the greatest repeatability in the mean bearing and modal dive depth. Individual‐level consistency was greatest for the proportion of benthic diving, total distance travelled, and trip duration. Indices of benthic foraging success and efficiency were positively influenced by consistency in the proportion of benthic diving, trip duration and dive rate but not influenced by consistency in bearing to most distal point, dive depth or foraging site fidelity. The results of the present study provide evidence of the benefits of consistency for individuals, which may have flow‐on effects at the population level.

## Graphical abstract

Low‐moderate level population‐level repeatability and individual‐level consistency were identified in female Australian fur seal foraging behaviors. Despite this, greater individual consistency in some behaviors was associated with greater foraging success and efficiency of benthic dives. The results of the present study provide evidence of the benefits of consistency for individuals, which may have flow‐on effects at the population level.

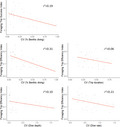

## 1 INTRODUCTION

The importance of individual‐ and population‐level differences in resource use and behavior has long been recognized (Foster, 1999). Foraging theory predicts that individuals adopt foraging strategies that maximize energy intake while minimizing energetic costs (Ydenberg et al., 1994). However, while ecological studies often treat all individuals within a group as effectively the same, substantial variation in foraging strategies can exist within populations (Bolnick et al., 2003). Such variation can result from differences in age, sex and/or morphology and can also be due to individual specialization, with individuals consistently exploiting a narrow behavioral, spatial or dietary niche over time (Bolnick et al., 2003). Individual specializations can arise even in populations typically regarded as generalists, whereby individuals specialize on different narrow niches within the overall population niche (i.e., Type 'B' generalists) (Araujo et al., 2011).

Specializations are widespread amongst wild animal populations (Bolnick et al., 2003) and are expected to be particularly prevalent among top‐order predators due to bottom‐up processes and resource competition (Estes et al., 2003). Such specializations are suggested to reduce intraspecific competition and increase reproductive success (Araujo et al., 2011). Furthermore, high degrees of individual specialization within Type 'B' generalist populations may lead to increased adaptability against environmental change, as individuals will likely respond differently to change (Tinker et al., 2008). As such, heterogeneity among foraging strategies may play an important role in the response of a species to environmental change.

Few studies have provided evidence of such benefits associated with foraging or dietary consistency in wild animal populations and the existing evidence is equivocal and differs between studies (e.g., Woo et al., 2008; Hatase et al., 2013). However, there is a larger body of evidence for the benefits of consistency in other behaviors, such as anti‐predator responses (Gutowsky et al., 2016) and migration (Jensen et al., 2020). The lack of consensus on whether foraging and dietary specialization is beneficial at the individual‐ or population‐level highlights the need for more studies into the ecological consequences of specialization (Araujo et al., 2011). Such knowledge is important for understanding how populations may respond to anticipated changes in their environment (Bolnick et al., 2003).

While many studies report improved reproductive success, fitness, and body condition resulting from foraging specializations (reviewed by Patrick & Weimerskirch, 2017), foraging specializations may also increase individual foraging success and efficiency through improved prey finding, handling and/or digestion (Estes et al., 2003) or due to reduced intraspecific competition (Bolnick et al., 2003). Foraging success and efficiency can have direct influences on reproductive success (Jeanniard‐du‐Dot et al., 2017). As such, factors that influence the foraging success and efficiency of individuals may have direct or indirect influences on offspring survival and population growth.

The Australian fur seal (*Arctocephalus pusillus doriferus*, AUFS) population is still considered to be recovering from the historic overexploitation of the commercial sealing era (1798–1825) (Kirkwood et al., 2010). Despite this, with a total population size of *ca* 120,000 individuals and female and male body masses of 75 kg and 229 kg, respectively, AUFS account for the largest marine predator biomass in south‐eastern Australia. Like most otariid seals (fur seals and sea lions), AUFS give birth to a single pup each year, followed by a lactation period of approximately 10 months (Arnould & Hindell, 2002). During this time, females alternate between periods ashore nursing (1–3 days) with foraging trips to sea (2–11 days) in which they adopt a central‐place foraging strategy (Arnould & Hindell, 2001). Due to the central‐place foraging strategy, the high level of resource competition in the area surrounding the colony is expected to lead to high interindividual variation and specialization in diet and foraging behavior (e.g., foraging trip duration and foraging site fidelity) (Baylis et al., 2015). Indeed, individual specialization is common among central‐place foraging marine predators (Baylis et al., 2015; Camprasse et al., 2017) and may provide a buffer for populations against environmental change and anthropogenic disturbance (Dias et al., 2011), particularly for Type 'B' generalist populations.

Female AUFS are almost exclusively benthic foragers, feeding on the shallow (depth 60–70 m) sea floor of Bass Strait (Arnould & Kirkwood, 2008). Consequently, female AUFS have a highly restricted foraging range and are, thus, particularly vulnerable to environmental change (Costa, 2007). This is of particular concern as the south‐east Australian marine region is currently one of the fastest warming areas in the world (Hobday & Pecl, 2014) with warming expected to continue over the coming decades (Hobday & Lough, 2011). Oceanic temperature increases in the region have already been linked to changes in the abundance and distribution of primary producers and prey species (Last et al., 2011). Such changes in prey availability can have significant consequences for marine predators within restricted foraging ranges.

Previous research on AUFS has revealed interindividual variation in foraging behavior, as well as intrinsic and extrinsic factors influencing this variation (Hoskins & Arnould, 2013; Hoskins et al., 2015; Kirkwood & Arnould, 2011). However, while stable isotope analyses have revealed a degree of individual dietary specialization in AUFS (Kernaléguen et al., 2015), there is little information on the degree of individual consistency in foraging behavior or habitat use in the species, nor the implications of such consistency. Knowledge of the degree and implications of behavioral consistency in AUFS is urgently needed in order to predict how the species may respond to the anticipated changes to the marine ecosystem.

Therefore, the aims of the present study were to: 1) investigate the level of population‐level (interindividual) repeatability and individual‐level (intraindividual) consistency in foraging behavior; 2) determine the degree of foraging site fidelity (the reuse of prior foraging areas); and 3) investigate if and how individual‐level consistency in foraging behavior influences the foraging success and efficiency of female Australian fur seals.

## 2 METHODS

### 2.1 Animal handling and instrumentation

**FIGURE 1:**
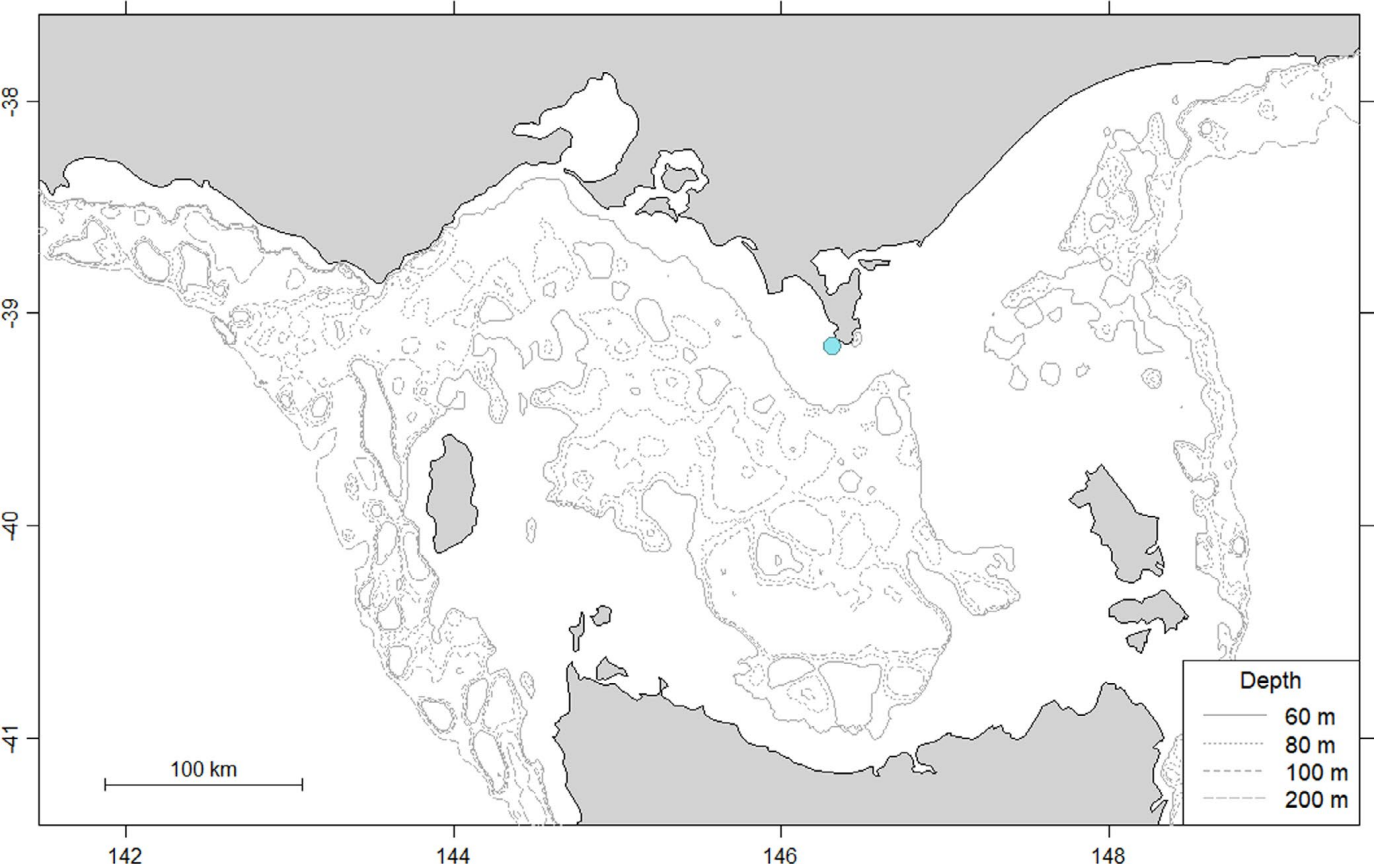
The location of the study site, Kanowna Island (blue circle), in south‐eastern Australia.

Field work was conducted on Kanowna Island (39°10’S, 146°18’E; Figure 1), central northern Bass Strait, south‐eastern Australia, during May‐August of 2006–2019. This island hosts the third largest breeding colony of AUFS, with an annual production of *ca* 3,400 pups (Kirkwood et al., 2010). Adult females (*n* = 34) observed suckling pups were selected at random and captured using a modified hoop net (Fuhrman Diversified, Seabrook, Texas, U.S.A). Upon capture, the animals were anesthetized with isoflurane gas delivered via a portable gas vaporizer (Stinger, Advanced Anaesthesia Specialists, Gladesville, NSW, Australia) and maintained on anesthesia for processing.

Individuals were weighed with an electronic suspension scale (± 0.5 kg) and morphometric measurements (± 0.5 cm) of standard length, flipper length, axillary girth, and axis length (nose to front of fore flipper along dorsal midline) were taken. A FastLoc GPS data logger (Sirtrack Ltd.) and dive behavior data logger (Mk09 or Mk10; Wildlife Computers Ltd.) were attached to the dorsal midline pelage just posterior to the scapula using a quick‐setting, two‐part epoxy resin (Accumix 268; Huntsman Advanced Materials). The GPS was programmed to sample location information when seals were at the surface at 10 min intervals and the dive behavior data loggers were programmed to sample at either 1 or 5 s intervals. A very high frequency (VHF) transmitter (Sirtrack Ltd.) was also glued to the pelage posterior to the other data loggers to facilitate relocation for recapture. Individually numbered plastic tags (Super Tags, Dalton, Woolgoolga, Australia) were then inserted into the trailing edge of each fore flipper before the animal was allowed to recover from anesthesia and resume normal behaviors.

Individuals were recaptured after at least three foraging trips to sea using the procedures outlined above and the data loggers were removed by cutting the fur beneath each device with a scalpel blade. Data from the data loggers were downloaded onto a portable computer in the field before being prepared for redeployment on other individuals.

### 2.2 Data processing

Data downloaded from the dive behavior loggers were zero‐offset corrected, to account for drift in the pressure readings, and dive metrics (time of dive, dive duration, maximum depth, and bottom time) were summarized using the *diveMove* package (Luque, 2019) within the R statistical environment (version 3.6.1; 29). A minimum dive threshold of 5 m was used to exclude surface activity for nonforaging purposes. For each foraging trip to sea, the proportion of benthic diving, proportion of dives during daylight hours (07:00–18:00 hr), dive rate (m·h^‐1^; calculated as the sum of total vertical distance travelled divided by the total time at sea; Boyd et al., 1994), and modal dive depth (m) were calculated. While AUFS are generally considered benthic foragers, pelagic foraging occurs in approximately 15%–22% of dives (Speakman et al., 2020). As benthic foraging has been shown to be more energetically costly than pelagic foraging (Costa, 1993), the proportion of benthic diving occurring during each foraging trip was calculated as an additional metric of foraging effort. The proportion of benthic diving was determined using the methods described in Hoskins et al. (Hoskins et al., 2015), whereby an index is derived representing the maximum depth achieved for each dive, weighted by the proportion of time spend at the bottom of each dive. This results in a bimodal density distribution of the index and dives to the left of the nadir (i.e., dives that are shallow with short bottom times, relative to other dives performed by the individual) are classified as pelagic and dives to the right of the nadir are classified as benthic (Figure S1).

A speed filter was applied to the GPS locations to account for erroneous data using a maximum travel speed of 6 m s^‐1^ and at sea movement tracks were linearly interpolated at 10 s intervals in the *adehabitatLT* package (Calenge, 2006) to allow merging of spatial information with dive behavior records by the nearest date and time. To account for individuals resting at haul‐out locations away from the breeding colony (Kirkwood & Arnould, 2011), a 1 km buffer around all known potential haul‐outs within Bass Strait was calculated. All GPS locations (at sea and on land) occurring within these buffer zones were excluded from further analyses and a foraging trip was defined as an individual leaving and returning to the breeding colony minus any time spent at the haul‐out locations. Because AUFS have been observed to spend several hours at a time in the water surrounding the colony for purposes other than foraging (e.g., thermoregulation; Arnould & Kirkwood, 2008), only continuous periods of ≥ 6 hr in the water in which at least one foraging dive occurred were considered a foraging trip, while haul‐out periods were defined as periods of ≥ 10 min out of the water.

For each foraging trip, total trip duration (h), total (vertical and horizontal) distance travelled (km), maximum straight‐line distance from the colony (km), and bearing (°) to the most distal location were calculated. These spatial metrics were then combined with the dive behavior metrics to provide a set of foraging behavior metrics per trip.

An index of consistency in spatial use was calculated for each individual. At sea movements on each foraging trip were overlaid with a 1 × 1 km grid using the *raster* package (Hijmans, 2020) and the total time spent diving per grid cell was calculated. The 95% kernel density estimate (using smoothing parameter, *h* = “href”) utilization distribution probabilities of time spent diving for each was then determined for each individual using *adehabitatHR* (Calenge, 2006) and *raster* packages. To measure the degree of overlap between foraging trips, Bhattacharyya's Affinity Index (Bhattacharyya, 1943) was calculated within each individual using the percentage overlap between kernels. The output value was termed the Foraging Site Fidelity Index (FSFI). This index measures the degree of overlap between each trip by multiplying the values with all pairwise combinations of trips (e.g., trip 1 × 2, trip 1 × 3, trip 2 × 3, etc.) per individual. The mean was calculated for each value resulting in an index score between 0 and 1, with 0 indicating no overlap and 1 indicating complete overlap in foraging areas between trips to sea.

To investigate the influence of foraging behavior consistency and FSFI on the foraging success and efficiency of individuals, indices of benthic foraging success and efficiency were calculated (Speakman et al., 2020). These indices were calculated using video‐validated relationships between descent rate, dive duration and the probability of individual AUFS successfully capturing prey (Volpov et al., 2016). The estimates derived in the previous step were then used to calculate the Foraging Trip Success Index (FTSI) for each foraging trip, representing the sum of each predicted prey capture success probability divided by the sum of each benthic dive duration for the foraging trip (Speakman et al., 2020). A Foraging Trip Efficiency Index (FTEI) was also calculated, representing the sum of prey capture success probabilities for each benthic dive divided by the dive rate (m h^‐1^) for benthic dives only as a measure of effort (Speakman et al., 2020). As only benthic dives were used in the validation process, foraging success, and efficiency calculations could only be applied to benthic dives in this analysis.

### 2.3 Statistical analyses

All statistical analyses were conducted in the R statistical environment (Team RCD, 2019). Data exploration followed the protocols outlined in Zuur, Ieno (Zuur et al., 2010). Prior to analysis, collinearity between predictor variables was assessed and, where *r* > 0.7 or <−0.7 (Zuur et al., 2009), one member of the pair was removed.

In order to determine the consistency in foraging behaviors between and within individuals, the optimal fixed effects structure (Table 2) for each response variable (maximum distance from the colony, total distance travelled, total trip duration, dive rate, modal dive depth, proportion of dives during daylight hours, and proportion of benthic diving) needed to be identified to account for intrinsic effects on foraging behavior. To determine the optimal fixed effects structure, Linear Mixed Effects (LME) models, fitted with restricted maximum likelihood (REML), were constructed in the *lme4* package (Bates et al., 2015) for the maximum distance from the colony, total distance travelled, total trip duration, dive rate, and modal dive depth. The proportion of benthic diving and proportion of dives occurring during daylight hours were fit using a GLMM with a binomial distribution and “logit” link and were otherwise fit in the same manner as the LME models.

All models were constructed using the “individual” as the random intercept for the model, to determine the variance associated with the individual, and morphometric variables as the fixed effects. Morphometric variables included standard length, flipper length, axillary girth, axis length, and mass. Assessment of collinearity resulted in the exclusion of mass and standard length from further analyses. Maximal models, including all remaining morphometric variables, were fitted with maximum likelihood and were inspected for outliers, heterogeneity of the residuals and the residual distribution. The total distance travelled, trip duration and dive rate were, consequently, cube‐root transformed to approximate a Gaussian distribution in the residuals. As no outliers or heterogeneity detected, model selection was conducted using the “dredge” function (*MuMIn* package; Barton, 2019) to determine the optimal fixed effects structure based on AICc and the difference in AICc (ΔAIC) with a threshold difference of < 4 (Burnham & Anderson, 2002). Models were refitted with REML to estimate model parameters to identify the influence of intrinsic factors on the foraging behavior (Zuur et al., 2009).

#### 2.3.1 Population‐level repeatability

Population‐level repeatability incorporates the variance in behavior associated with the interindividual component (i.e., variation between individuals) and the intraindividual component (i.e., variation between trips of the same individual), and represents the proportion of the total variance that is explained by the interindividual level.

Once optimal models, as selected above, were determined, the population‐level repeatability (R) for each variable was calculated as follows:

$$R=\frac{\sigma_{\alpha }^{2}}{\sigma_{\alpha }^{2}+\sigma_{\in }^{2}},$$

where $\sigma_{\alpha }^{2}$ is the interindividual variability and $\sigma_{\in }^{2}$ is the intraindividual variability (residual error; Dingemanse & Dochtermann, 2013). Repeatability of each behavior was calculated using the *rptR* package (Stoffel et al., 2017). All normally distributed response variables were fitted with a Gaussian distribution using the “rpt*”* function, while proportional data were fitted with a “logit” link using the “rptProportion” function. Permutation tests and bootstrapped 95% confidence intervals were used for Gaussian response variables (rptProportion does not calculate confidence intervals) to determine the significance of repeatability estimates. Each foraging behavior variable was run as a separate model, including the optimal fixed effects structure identified using LME and GLMM models. Where fixed effects were included, the repeatability estimates were considered adjusted repeatabilities ($R_{\text{adj}}$), accounting for variance associated with the included morphometrics (i.e., representing the repeatability as if all measurements were for individuals of the same morphometric measurements) (Nakagawa & Schielzeth, 2010).

Repeatability estimates range from 0 to 1. Following the definitions in Harris et al. (Harris et al., 2014), behaviors with repeatability estimates of 0–0.25 were classified as having low repeatability, estimates of 0.25–0.5 as having considerable repeatability, estimates of 0.5–0.75 as having moderate repeatability, while estimates of > 0.75 as being highly repeatable. As repeatability estimates are calculated using the inter and intraindividual variance, the population‐level repeatability will be inverse to the individual‐level repeatability.

#### 2.3.2 Individual‐level repeatability and individual consistency

Individual‐level repeatability for each foraging behavior was calculated as:

$R^{\prime }=1‐R,$ where R is the population‐level repeatability. As with the population‐level repeatability, the $R^{\prime }$ ranges from 0 (low variability) to 1 (high variability). As with the population‐level repeatability, repeatability estimates were considered adjusted where fixed effects were included in the model.

Having established that the population has moderate to high individual‐level variability in foraging behavior, coefficients of variation (CV) were calculated as the standard deviation divided by the mean for each of the foraging behavior metrics for each individual to quantify how consistent each individual was in foraging behavior and spatial use. As bearing is a circular statistic, the standard deviation for bearing was calculated using the *circular* package (Agostinelli & Lund, 2011) in place of the CV. Smaller CV values are indicative of greater individual consistency, while larger values indicate lower individual consistency. Additionally, CV values for the FTSI and FTEI for each individual were calculated to provide a measure of variability in foraging success and efficiency.

To investigate how individual variation, both in foraging behavior and foraging site fidelity, influences the foraging success and efficiency, or variability within these indices, four linear models (LM) were constructed using the *nlme* package (Pinheiro et al., 2019) with the CV for each of the foraging behavior metrics and the FSFI as predictor variables for the FTSI, FTEI and the variability in FTSI and FTEI (as measured through CVs). The CVs for the total distance and total horizontal distance travelled were excluded from the LMs due to correlation with the trip duration. The optimal model structure was selected using the model selection process described above model estimates were derived. Unless otherwise indicated, results are reported as Mean ± SE.

## 3 RESULTS

Data were obtained from a total of 34 individuals that completed a minimum of 3 foraging trips (Median = 5.5), totally 236 trips. These individuals had body masses and standard lengths of 70.4 ± 0.7 kg and 151.4 ± 0.5 cm, respectively. Flipper length, axis length, and axillary girth measurements averaged 42.5 ± 0.1 cm, 63.2 ± 0.3 cm and 99.2 ± 0.4 cm, respectively (Table 1). Individual foraging trips lasted 103.5 ± 3.7 hr on average, with individuals travelling 81.1 ± 3.3 km from the colony. The mean bearing was 203.5º (*SD*: 49.1º), with most foraging trips occurring within central Bass Strait, south‐west of the colony (Figure 1). The total distance travelled during foraging trips averaged 390.2 ± 1.4 km. Mean foraging dive duration was 155.3 ± 3.6 s, for mean modal dive depths of 62.2 ± 2.0 m, with individuals achieving dives rates of 1,117.3 ± 105.5 m h^‐1^. On average, 75.6 ± 1.7% of dives were benthic and 44.1 ± 1.7% of active foraging occurred during daylight hours (Table S1).

**TABLE 1:** Summary morphometrics for lactating female Australian fur seals from the Kanowna Island breeding colony, Bass Strait, Australia, instrumented between 2006 and 2019

Body size measurement	Mean ± SE	Range
Mass (kg)	70.4 ± 0.7	48.5–91.5
Standard length (cm)	151.4 ± 0.5	133.0–167.0
Flipper length (cm)	42.5 ± 0.1	37.0–48.5
Axis length (cm)	63.2 ± 0.3	53.0–72.5
Axillary girth (cm)	99.2 ± 0.4	78.0–112.5

### 3.1 Population‐level repeatability

**FIGURE 2:**
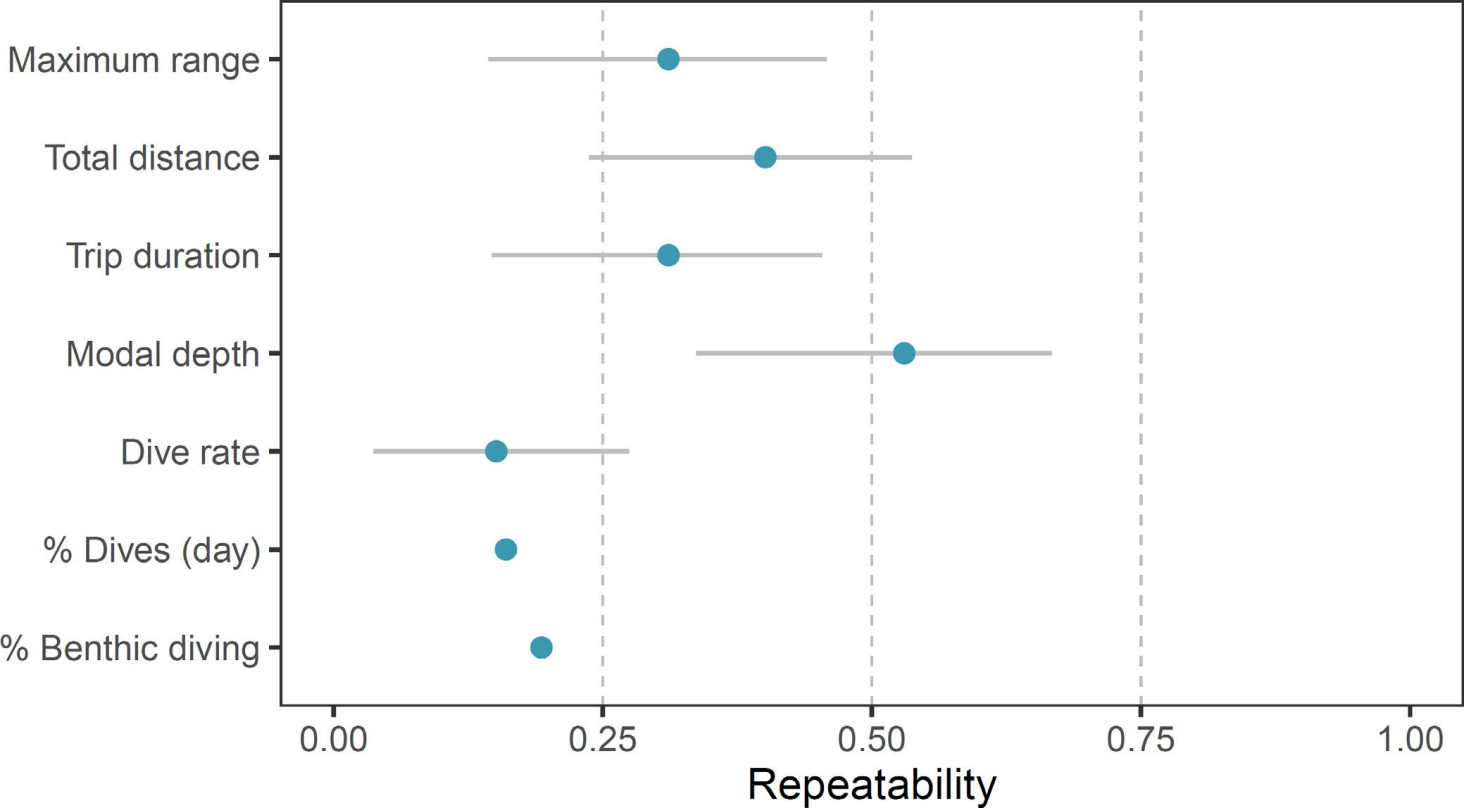
Repeatability estimates for foraging behaviors of adult female Australian fur seals with 95% CI. Repeatability estimates for “% Dives (day)” and “% Benthic diving” were calculated using the rptProportion function (rptR package), which does not provide confidence intervals around estimates

The inclusion of fixed effects was supported in all models except those explaining the proportion of dives during daylight hours and dive rate (Table 2). Repeatability estimates for all foraging behavior metrics were significant, but the strength of repeatability varied considerably among behaviors (*R* = 0.15–0.40 and *R*_adj_ = 0.19–0.53; Figure 2). There was considerably greater repeatability in spatial variables than in those describing dive behavior, with the exception of modal dive depth, which was the most repeatable behavior (Figure 2).

**TABLE 2:** Summary of the optimal models selected for the repeatability analysis for adult female Australian fur seals, indicating the significance of the fixed effects, when the optimal model included fixed effects

Response variable	Models	*df*	AIC	logLik	*p*	Covariate	Est.	SE	*t*	95% CI
Maximum distance from the colony (km)	Range ~ 1 + (1 | ID)	3	2,544.90	−1269.5						
	Range ~ Axis length + Axillary girth + Flipper length + (1 | ID)	6	2,539.70	−1263.9	0.011	(Intercept)	219.03	112.01	1.96	7.4–430.9
						Axis length	−1.75	1.19	−1.47	−4.0–0.5
						Axillary girth	2.44	0.88	2.77	0.8–4.1
						Flipper length	−6.26	2.48	−2.52	−11.0–−1.6
Total distance travelled (km)	Total distance ~ 1 + (1 | ID)	3	787.05	−390.5		(Intercept)	7.12	0.17	42.20	6.8–7.5
Trip duration (h)	Trip duration ~ 1 + (1 | ID)	3	602.81	−298.4		(Intercept)	4.57	0.10	45.08	4.4–4.8
Modal dive depth (m)	Depth ~ 1 + (1 | ID)	3	2,264.60	−1129.3						
	Depth ~ Axis length + Axillary girth + (1 | ID)	5	2,261.60	−1125.8	0.030	(Intercept)	144.16	96.64	1.49	−41.9–330.4
						Axis length	2.14	0.92	2.34	0.4–3.9
						Axillary girth	−1.32	0.64	−2.06	−2.6–−0.1
Dive rate (m·h^−1^)	Dive rate ~ 1 + (1 | ID)	3	1,263.40	−628.7		(Intercept)	9.24	0.31	30.11	8.6–9.9
Proportion of dives during daylight hours (PDD)	PDD ~ 1 + (1 | ID)	2	307.69	−151.9		(Intercept)	−0.70	0.22	−3.12	−1.2–−0.3
Proportion of benthic diving (PBD)	PBD ~ 1 + (1 | ID)	2	197.56	−96.8						
	PBD ~ Axis length + Axillary girth + (1 | ID)	4	192.21	−92.1	0.009	(Intercept)	1.72	7.30	0.24	−12.7–16.9
						Axis length	0.20	0.07	2.92	0.1–0.4
						Axillary girth	−0.08	0.05	−1.60	−0.2–0.0

Of the dive behavior metrics, only modal dive depth and the proportion of benthic diving were significantly influenced by morphometrics (Table 2). A positive correlation was found between axis length both the proportion of benthic diving (GLMM: Est = 0.20, *z* = 2.92) and the modal dive depth (LME: Est = 2.14, *t* = 2.34). Modal dive depth was also negatively associated with the axillary girth (LME: Est = −1.32, *t* = −2.06). Maximum distance from colony was positively correlated with axillary girth (LME: Est = 2.44, *t* = 2.77) and negatively correlated with flipper length (LME: Est = −6.26, *t* = −2.52).

### 3.2 Individual‐level repeatability and consistency

**FIGURE 3:**
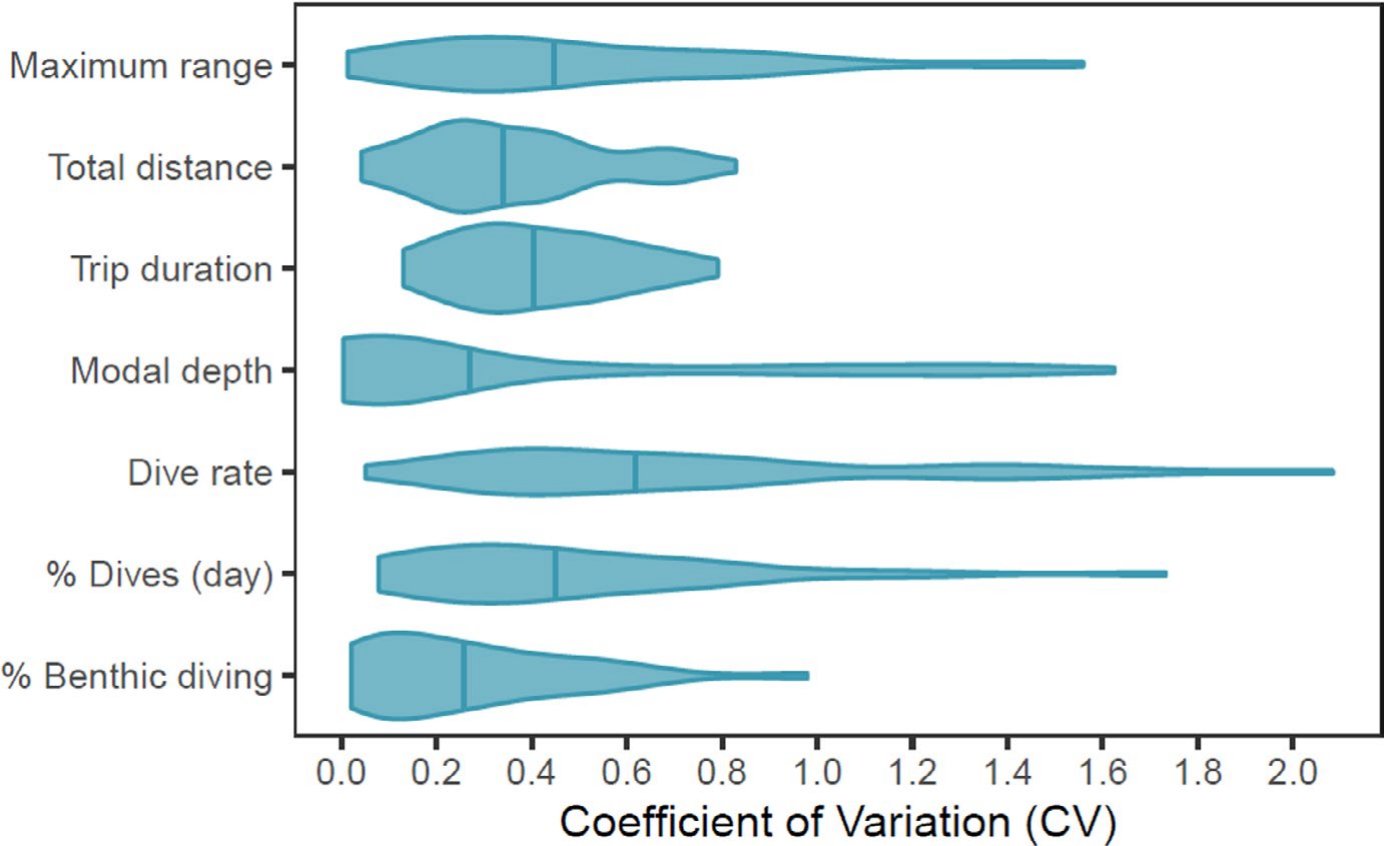
Individual‐level consistency in foraging behaviors of adult female Australian fur seals represented by the coefficient of variation (CV)

The variance associated with the individual contributed > 60% of the variance observed (i.e., the individual‐level repeatability was > 0.60) for each of the foraging behaviors with the exception of the modal dive depth, which was equally influenced by the inter and intraindividual components (Table 3). The wide range of CV values for each of the foraging behaviors (Figure 3) indicates substantial variation between individuals in their level of consistency. The highest degree of consistency was observed for the total distance travelled during a foraging trip, the trip duration and the proportion of benthic diving. A lower degree of consistency was observed for the maximum distance from colony, modal dive depth and the proportion of diving during daylight hours (Figure 3), while the greatest degree of variation was observed for dive rate.

**TABLE 3:** Variance ($\sigma^{2}$) explained at the individual‐level for foraging behaviors in female Australian fur seals. Variances were obtained using the optimal fixed effects structure identified in Table 2

Response variable	$\sigma^{2}$
Maximum distance from the colony (km)	68.9%
Horizontal distance travelled (km)	58.7%
Total distance travelled (km)	59.9%
Trip duration (h)	68.9%
Modal dive depth (m)	47.0%
Dive rate (m·h^−1^)	84.9%
Day dives (%)	84.0%
Benthic diving (%)	80.7%

**FIGURE 4:**
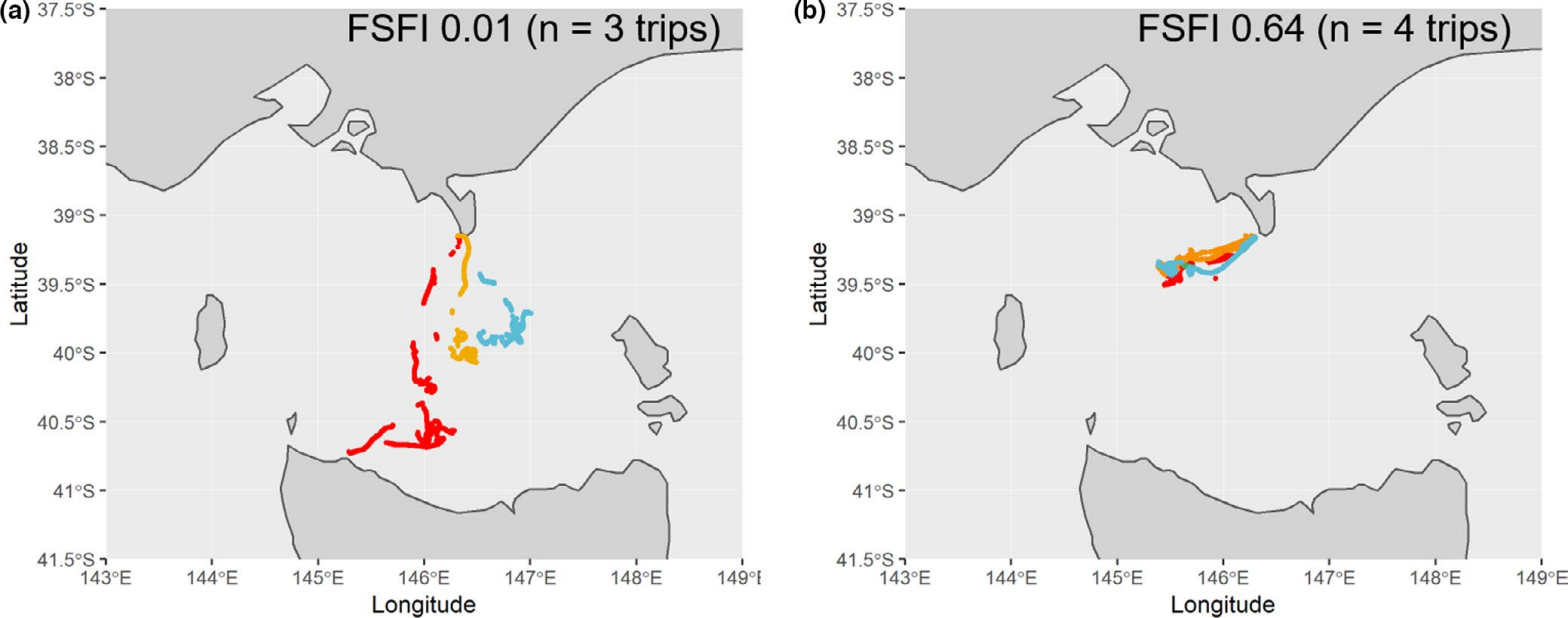
GPS tracks from consecutive foraging trips representing female Australian fur seals with A) low and B) high foraging site fidelity as measured by the Foraging Site Fidelity Index (FSFI). Colors indicate different foraging trips

Individuals were highly variable in their spatial habitat use (Median bearing *SD*: 56.2°; Median FSFI: 0.18; Table S1). However, there was a considerable range in FSFI values (0.01–0.64; Figure 4), with four individuals exhibiting moderate site overlap (FSFI > 0.50; Table S1). Substantial variation in bearing to most distal point was also observed (12.0°‐163.9°; Table S1).

### 3.3 Influence of individual‐level consistency on foraging success and efficiency

**FIGURE 5:**
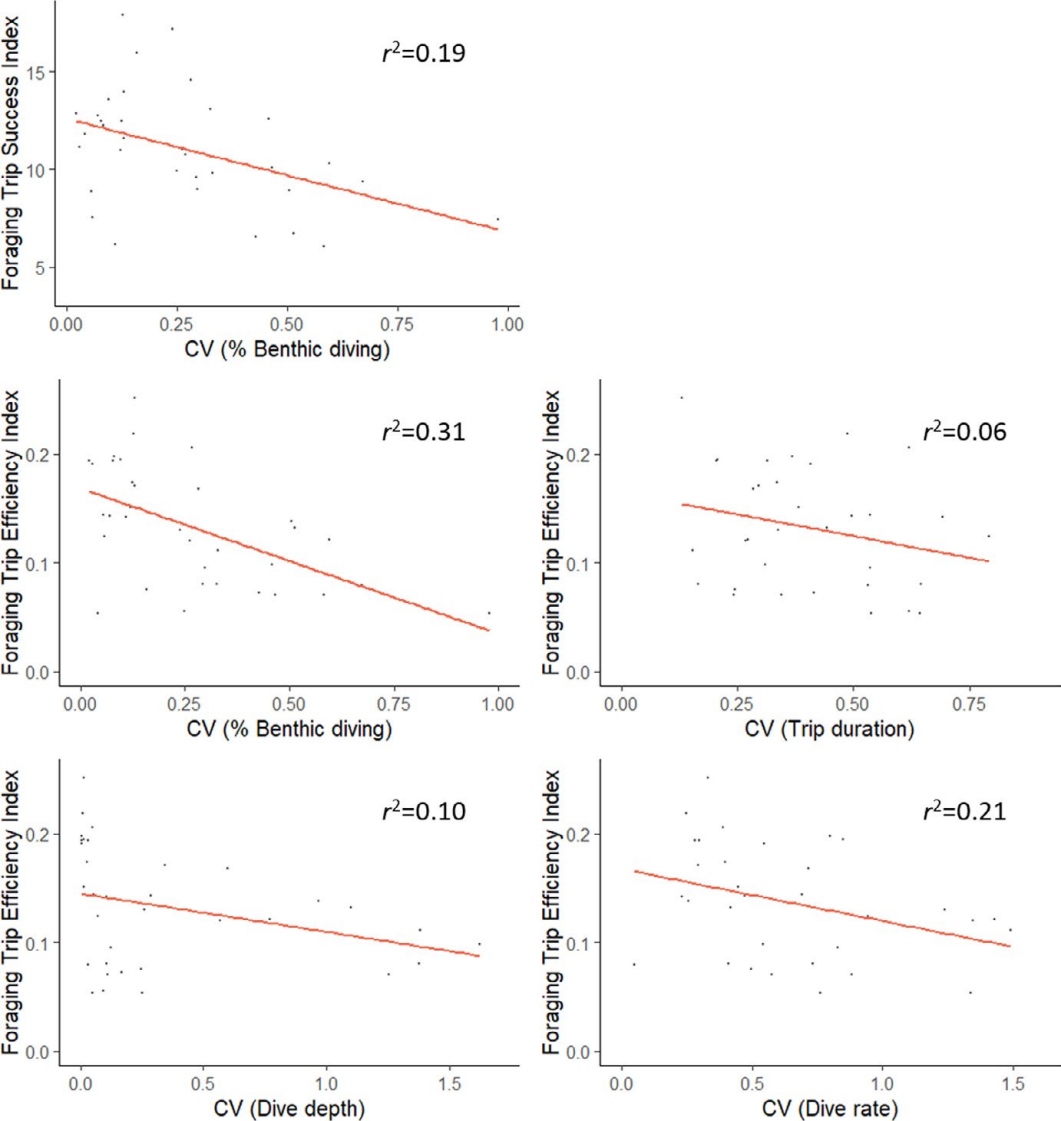
Significant influences of individual‐level consistency (measured using the coefficient of variation, CV) in foraging behavior on the benthic foraging success and efficiency in female Australian fur seals. Significance here refers to relationships whose 95% CI do not cross zero. Optimal models and nonsignificant relationships can be seen in Table 4

The FTSI and FTEI, representing the benthic foraging success and efficiency, ranged from 6.05–17.88 and 0.05–0.25, respectively. Low variability in FTSI (CV = 0.06–0.42) and moderate variability in FTEI (CV = 0.05–1.09) was observed. The most parsimonious model investigating the influence of foraging behavior consistency on the FTSI included the CVs for the proportion of benthic diving and trip duration (Table 4). The FTSI was negatively correlated with both the variability in the proportion of benthic diving (LM: Est. = −5.47, *t* = −2.66) and for the variability in trip duration (LM: Est. = −4.90, *t* = −1.85) (Table 4; Figure 5). In addition, the optimal model explaining the variability in FTSI (i.e., the CV of the FTSI) also included the variability in trip duration, as well as the variability in dive rate (Table 4). Greater variability in these foraging metrics were associated with increased variability in the FTSI (LM: Est = 0.15, *t* = 2.19; and Est = 0.07, *t* = 2.68, respectively) (Table 4).

**TABLE 4:** Summary of the optimal models explaining the influence of individual consistency in foraging behavior of adult female Australian fur seals on the benthic foraging success (FTSI) and efficiency (FTEI) indices, and the variability within these indices

Model	Formula	*df*	AIC	L‐ratio	*p*	*r* ^2^	Covariate	Est	SE	*t*	95% CI
FTSI	FTSI ~ 1	2	173.97								
	FTSI ~ CV (% Benthic diving) + CV (Trip duration)	4	166.89	11.09	0.004	0.28	(Intercept)	14.25	1.27	11.25	11.6–16.9
							CV (% Benthic diving)	−5.47	2.06	−2.66	−9.8–−1.2
							CV (Trip duration)	−4.90	2.65	−1.85	−10.4–0.6
FTEI	FTEI ~ 1	2	−99.25								
	FTEI ~ CV (% Benthic diving) + CV (Depth) + CV (Dive rate) + CV (Trip duration)	5	−119.81	28.55	<0.001	0.57	(Intercept)	0.23	0.02	10.70	0.2–0.3
							CV (% Benthic diving)	−0.11	0.03	−3.36	−0.2–−0.0
							CV (Depth)	−0.03	0.02	−1.64	−0.1–0.0
							CV (Dive rate)	−0.04	0.01	−2.93	−0.1–−0.0
							CV (Trip duration)	−0.10	0.04	−2.40	−0.2–−0.0
Variability in FTSI	CV (FTSI) ~ 1	2	−73.05								
	CV (FTSI) ~ CV (Dive rate) + CV (Trip duration)	4	−81.05	12.00	0.003	0.30	(Intercept)	0.04	0.03	1.20	−0.0–0.1
							CV (Dive rate)	0.07	0.02	2.68	0.0–0.1
							CV (Trip duration)	0.15	0.07	2.19	0.0–0.3
Variability in FTEI	CV (FTEI) ~ 1	2	1.44								
	CV (FTEI) ~ CV (% Benthic diving) + CV (Dive rate) + CV (Trip duration)	5	−11.79	19.23	<0.001	0.43	(Intercept)	0.00	0.10	−0.02	−0.2–0.2
							CV (% Benthic diving)	0.23	0.15	1.55	−0.1–0.5
							CV (Dive rate)	0.17	0.07	2.53	0.0–0.3
							CV (Trip duration)	0.66	0.19	3.43	0.3–1.0

The variability in the proportion of benthic diving, trip duration and maximum range from the colony were included in the optimal model explaining the FTEI (Table 4). The FTEI exhibited a negative relationship with each of these explanatory variables, with significant influences observed for the variability in dive rate (LM: Est = −0.04, *t* = −2.93) and the variability in the proportion of benthic diving (LM: Est = −0.11, *t* = −3.36) (Table 4; Figure 5). The variability in FTEI was positively influenced by the variability in dive rate (LM: Est = 0.17, *t* = 2.53) and variability in the trip duration (LM: Est = 0.66, *t* = 3.43) (Table 4).

## 4 DISCUSSION

The present study detected low to moderate degrees of population‐level repeatability in the foraging behavior of female AUFS, with greater repeatability in spatial metrics than in diving metrics. With the exception of modal dive depth, the majority of the variance observed in foraging behaviors was explained at the intraindividual level. The level of individual‐level consistency varied widely between individuals for each of the foraging behavior metrics, with the least variation observed for the proportion of benthic diving, total distance travelled and trip duration. Similarly, while foraging site fidelity was generally low, there was substantial variation between individuals. Importantly, this study found that greater individual‐level consistency in foraging behavior was associated with greater success and efficiency, and greater consistency in efficiency, in benthic foraging.

### 4.1 Population‐level repeatability and individual‐level consistency in foraging behavior

Some studies suggest that benthic foraging marine species should display greater degrees of foraging specialization than pelagic foraging species, as benthic foragers can utilize sea‐floor features to return to previously profitable foraging areas (Mattern et al., 2007) and benefit from more predictable prey resources (Arnould & Costa, 2006). However, studies have reported varying degrees of foraging specialization between populations or colonies in both benthic and pelagic foraging species (Kuhn et al., 2014; McHuron et al., 2018). Specializations are also influenced by ecological opportunity, and the differences observed in the level of specialization across studies may also reflect differing degrees of prey abundance and/or diversity or habitat size (Araujo et al., 2011).

Several studies have documented inter or intraindividual variation in habitat use, diving behavior, and diet across foraging trips in adult female Australian fur seals (Arnould et al., 2011; Hoskins et al., 2015; Kernaléguen et al., 2016). In the present study, low to moderate degrees of foraging behavior repeatability were observed between foraging trips at the population level, with the modal dive depth being the most repeatable behaviors assessed. However, individuals were typically more variable in modal dive depth than other foraging behaviors at the individual level. Bass Strait has a fairly uniform bathymetry (mean depth 60–70 m) and, as female AUFS are predominantly benthic foragers, female AUFS displayed a high level of population‐level repeatability in modal dive depth. The individual‐level variation in modal dive depth that was observed may reflect usage of different areas of Bass Strait that have shallower or deeper sea‐floor depths or may be a result of individuals opportunistically targeting pelagic prey at a greater frequency during some foraging trips.

Moderate population‐level repeatability was found for the maximum distance from colony, total distance travelled and trip duration across foraging trips. Similarly, moderate to high population‐level repeatability in maximum distance to colony was observed in California sea lions, *Zalophus californianus* (McHuron et al., 2018), and Imperial shags, *Phalacrocorax atriceps* (Harris et al., 2014). However, this similarity did not extend to the trip duration and total distance travelled was not assessed in these studies. Further, individual‐level consistency in trip duration was lower than for the maximum distance to colony in California sea lions (McHuron et al., 2018). Contrastingly, individuals in the present study were more consistent in their total distance travelled and their trip duration than they were for the maximum distance from the colony. Both trip duration and distance travelled are impacted by the foraging location but may also be influenced by periods of reduced or improved prey availability (Croxall et al., 1999). The results of the present study suggest female AUFS respond to changes in prey availability by altering the distance they travel from the colony, rather than modifying the overall energy expenditure of the trip under potentially uncertain prey availability.

Intrinsic factors, such as age and morphology, have also been associated with variability in foraging behavior (Bolnick et al., 2003). However, there was little influence of morphology on foraging behaviors in the present study. In AUFS, standard length and mass are potential indicators of age (Arnould & Warneke, 2002) and, in the present study, these morphometrics were both correlated with axillary girth. Interestingly, larger, presumably older, individuals had greater variability in trip duration and total distance travelled. While this may appear counter‐intuitive, the increased variability associated with larger/older individuals may indicate that the individuals may be responding better to differing environmental conditions or variations in prey distributions.

While AUFS are generally considered benthic foragers, with > 75% of recorded dives being to the sea floor (Speakman et al., 2020), population‐level repeatability in the proportion of benthic diving was low. However, the proportion of benthic diving was the most consistent, at the individual level, of the foraging behaviors assessed. This suggests that individuals may specialize in the level of benthic diving that they perform (i.e., individuals who forage pelagically more often tend to do so consistently). Indeed, there were several individuals that displayed high levels of pelagic diving across the majority of their trips to sea. These individuals also had a greater proportion of dives during the night, suggesting that they may have been making use of diel vertically migrating prey (Croxall et al., 1985). A similar but inverse pattern has been reported in the conspecific Cape fur seals (*A. p. pusillus*) at the Kleinsee seal colony, South Africa (Kirkman et al., 2019). Like most fur seals, Cape fur seals are predominantly pelagic foragers (Arnould & Costa, 2006). However, some individuals display greater proportions of benthic diving (as much as 80%) than the population average (16.4 ± 3.2%) (Kirkman et al., 2019), which the authors suggest may be a result of inadequate pelagic prey availability.

Low population‐level repeatability was also detected for the proportion of diving occurring during daylight hours and for dive rate. However, over a third of the individuals in the study dived mostly at night. These individuals typically exhibited greater proportions of pelagic diving. This suggests that some individuals are likely specialized toward pelagic night diving, likely utilizing the diel vertical migration of pelagic bait fish known to occur in the AUFS diet (Williams & Pullen, 1993). Knox et al. (2017) reported the use of diel strategies in male AUFS with some individuals being predominantly nocturnal foragers, some being diurnal foragers, while others showed no preference, consistent with the observations in the present study.

While individuals in the present study generally displayed low foraging site fidelity, there was a wide range in the level of site fidelity observed between individuals with four individuals exhibiting moderate‐high site overlap. The average foraging site fidelity was consistent with that detected in adult male AUFS (Knox et al., 2018), and was further supported by the variation observed in the bearing to most distal point. This contrasts with the high foraging site fidelity reported for benthic foraging Australian sea lions, *Neophoca cinerea*, (Lowther et al., 2012), New Zealand sea lions, *Phocarctos hookeri*, (Chilvers, 2008) and South American sea lions, *Otaria flavescens*, (Baylis et al., 2017).

The low foraging site fidelity in the present study could be an artifact of the duration of the sampling period, with the considerable variation in bearing observed reflecting individuals accessing multiple foraging areas on a rotational basis to allow for depletion and recovery of prey at the various sites. Under such a scenario, if more foraging trips were recorded, a higher foraging site fidelity may have been observed. However, this was not typically the case as individuals in the present study tracked for ≥ 10 foraging trips had an average FSFI of 0.27. Given that individuals in the present study were deployed for up to four months, this could indicate that rates of patch replenishment are low for benthic prey such that it is not economical for individuals to return within such timeframes to an area. Indeed, one of the main prey species for AUFS are benthic leatherjacket fish (family Monocanthidae) (Arnould et al., 2011), which reach maturity at 1–2 years (Visconti et al., 2018), such that depletion of mature adults may lead to a lagged replenishment of prey. The timescales over which individual specializations are assessed are known to influence the degree of specialization detected (Kernaléguen et al., 2016) and are likely to be influenced by the temporal consistency of prey resources. Therefore, future studies should focus on instrumenting individuals over multiple years to determine whether individual consistency in AUFS foraging behavior differs across short to long timescales or with changes in environmental conditions.

### 4.2 Influence of individual consistency on benthic foraging success and efficiency

Foraging success and efficiency have been shown to directly influence weaning success and/or offspring survival in marine predators (Jeanniard‐du‐Dot et al., 2017). Consequently, it is important to understand factors influencing foraging success and efficiency for predicting how a population may respond to threats, such as environmental change and human‐wildlife conflict. Foraging success and efficiency can be influenced by a range of intrinsic (Sutton et al., 2020) and extrinsic factors (Carroll et al., 2016). Indeed, benthic foraging success and efficiency in AUFS appear to be influenced by both intrinsic and extrinsic factors (Speakman et al., 2020). The levels of benthic foraging success and efficiency detected in the present study are consistent with those previously reported in the study population (Speakman et al., 2020).

While individual specializations have been reported in a range of taxa (Dall et al., 2012), evidence for behavioral consistency conferring benefits to individuals or populations has been rarely documented and is equivocal. For example, whereas specialized black‐browed albatrosses (*Thalassarche melanophris*) with a narrow niche width were more successful breeders than generalists within the same population (Patrick & Weimerskirch, 2017), some studies have reported no apparent benefit of consistency on the population (Woo et al., 2008).

The results of the present study provide evidence that behavioral consistency may be beneficial for female AUFS through improvements in success and efficiency of benthic foraging dives. Benthic dives account for the majority of dives in female AUFS and, therefore, improvements in the success and efficiency of benthic dives may have significant benefits for individuals over an entire foraging trip and/or at longer timescales. Individuals that were more consistent in the proportion of benthic diving had greater benthic foraging success. Additionally, the foraging efficiency of benthic dives was greater when individuals were more consistent in their trip duration, dive rate and the proportion of benthic diving. Furthermore, individuals were more consistent in their level of benthic foraging success and efficiency across trips when they were more consistent in their foraging trip durations and dive rates.

The almost exclusively benthic foraging behavior of AUFS is thought to be due to the low primary productivity of the Bass Strait region leading to an unpredictable and low availability of pelagic prey (Gibbs et al., 1986). Furthermore, while benthic foraging is considered more energetically costly than pelagic foraging (Arnould & Costa, 2006), the profitability of benthic prey has been shown to outweigh the reduction in energetic costs associated with pelagic prey in the benthic foraging AUFS (Meyers, 2019). The high variation in the proportion of benthic diving exhibited by some individuals, therefore, may be a result of inexperience or individuals trying to make use of changes in pelagic prey availability. The influence of trip duration on the benthic foraging efficiency could also be an artifact of lower experience, as more experienced individuals may be better able to maximize their foraging efficiency by knowing where and when to forage (Switzer, 1993). Alternatively, it could reflect that some individuals “hedge their bets” by moving between potential foraging areas, thus varying trip durations, rather than returning to areas that had previously resulted in success. More plausibly, this influence of trip duration on foraging efficiency may reflect individuals finding highly profitable prey and/or finding prey more rapidly during some foraging trips, and not during others. In addition, individuals may move away from previously profitable foraging locations due to depletion of prey resources (Charnov, 1976).

Furthermore, the influence of variability in dive rate on foraging efficiency and the variability in success and efficiency suggests that individuals who utilize different foraging strategies do not benefit from being generalists. However, flexibility in foraging behavior is likely adaptive, allowing individuals to adjust to changes in environmental conditions and competition. While the results of the present study indicated that individuals benefited from more consistent behavior, the persistence of generalists within populations may provide a buffer for the species against environmental change (Araujo et al., 2011). Individuals in the present study were tracked over short timeframes (i.e., weeks/months) but individuals need to be adaptable to environmental change over much longer timeframes (i.e., their lifetime), which may result generalist behaviors persisting in the population. The presence of a variety of specialists within the generalist population (Type 'B' generalists) may also provide a similar buffer by reducing intraspecific competition and allowing for adaptation to environmental change (Araujo et al., 2011).

In summary, the present study demonstrates low to moderate population‐level repeatability in foraging behavior in female AUFS. Greater repeatability was typically observed in spatial metrics than in dive‐based metrics. Behaviors that were moderately repeatable at the population‐level, such as dive depth, were also moderately highly consistent at the individual‐level. Furthermore, female AUFS appeared to benefit from greater consistency in foraging behavior, particularly in the proportion of benthic diving, with improved benthic foraging success and efficiency. However, the high level of population‐level variability in foraging behaviors may better enable female AUFS to respond to changes in their environment. This is particularly important under future climate predictions that anticipate rapid warming, salinification, deoxygenation and sea‐level rise, as well as increasing frequency and severity of storm systems (Hobday & Lough, 2011). Prey availability, distribution and diversity are likely to be greatly impacted under anticipated environmental change through changes in nutrient supply and ocean circulation (Doubleday et al., 2013), which is likely to have significant flow‐on effects for higher trophic‐levels (Frederiksen et al., 2006).

## CONFLICT OF INTEREST

The authors declare no competing or financial interests.

## AUTHOR CONTRIBUTIONS

CNS: Data curation lead; Formal analysis (lead); Investigation (lead); Methodology (lead); Visualization (lead); Writing‐original draft (lead); Writing‐review & editing (equal). STL: Data curation (supporting); Formal analysis (supporting); Methodology (supporting). ECMC: Formal analysis (supporting); Methodology (supporting); Writing‐review & editing (supporting). AJH: Methodology (supporting); Validation (supporting); Writing‐review & editing (supporting). MAH: Funding acquisition (equal); Resources (equal); Writing‐review & editing (supporting). DPC: Funding acquisition (equal); Resources (equal); Writing‐review & editing (supporting). JPYA: Funding acquisition (lead); Investigation (supporting); Methodology (supporting); Project administration (lead); Resources (lead); Supervision (lead); Writing‐review & editing (equal).

## ETHICAL APPROVAL

All work was carried out with the approval of the Deakin University Animal Ethics committee and under Department of Sustainability and Environment (Victoria, Australia) wildlife research permits (10,000,187,10,000,706,10,001,143,10,001,672,10,005,362, and10005848).

## Supporting information

Supplementary Material

## Data Availability

Data used in the analysis is available in Dryad at https://doi.org/10.5061/dryad.np5hqbzsb

## References

[ece37337-bib-0001] Agostinelli, C. , & Lund, U. (2011) R package circular: circular statistics. R package version 0.4‐3. https://cran.r‐project.org/package=circular

[ece37337-bib-0002] Araujo, M. S. , Bolnick, D. I. , & Layman, C. A. (2011). The ecological causes of individual specialisation. Ecology Letters, 14(9), 948–958. 21790933 10.1111/j.1461-0248.2011.01662.x

[ece37337-bib-0003] Arnould, J. P. Y. , Cherel, Y. , Gibbens, J. , White, J. G. , & Littnan, C. L. (2011). Stable isotopes reveal inter‐annual and inter‐individual variation in the diet of female Australian fur seals. Marine Ecology Progress Series, 422, 291–302. 10.3354/meps08933

[ece37337-bib-0004] Arnould, J. P. Y. , & Costa, D. (2006). Sea lions in drag, fur seals incognito: insights from the otariid deviants. Sea Lions of the World: conservation and research in the 21st Century Fairbanks, Alaska. p. 309‐323.

[ece37337-bib-0005] Arnould, J. P. Y. , & Hindell, M. A. (2001). Dive behaviour, foraging locations, and maternal‐attendance patterns of Australian fur seals (*Arctocephalus pusillus doriferus*). Canadian Journal of Zoology, 79(1), 35–48.

[ece37337-bib-0006] Arnould, J. P. Y. , & Hindell, M. A. (2002). Milk consumption, body composition and pre‐weaning growth rates of Australian fur seal (*Arctocephalus pusillus doriferus*) pups. Journal of Zoology, 256(3), 351–359.

[ece37337-bib-0007] Arnould, J. P. Y. , & Kirkwood, R. (2008). Habitat selection by female Australian fur seals (*Arctocephalus pusillus doriferus*). Aquatic Conservation: Marine and Freshwater Ecosystems, 17(S1), S53–S67.

[ece37337-bib-0008] Arnould, J. P. Y. , & Warneke, R. M. (2002). Growth and condition in Australian fur seals (*Arctocephalus pusillus doriferus*) (Carnivora : Pinnipedia). Australian Journal of Zoology, 50(1), 53–66. 10.1071/ZO01077

[ece37337-bib-0057] Barton, K. (2019) MuMIn: Multi‐Model Inference. R package version 1.43.6. https://cran.r‐project.org/package=MuMIn

[ece37337-bib-0009] Bates, D. , Mächler, M. , Bolker, B. , & Walker, S. (2015). Fitting linear mixed‐effects models using *lme4* . Journal of Statistical Software, 67(1), 1–48.

[ece37337-bib-0010] Baylis, A. M. , Orben, R. A. , Arnould, J. P. , Peters, K. , Knox, T. , Costa, D. P. , & Staniland, I. J. (2015). Diving deeper into individual foraging specializations of a large marine predator, the southern sea lion. Oecologia, 179(4), 1053–1065. 26323982 10.1007/s00442-015-3421-4

[ece37337-bib-0011] Baylis, A. M. M. , Orben, R. A. , Costa, D. P. , Tierney, M. , Brickle, P. , & Staniland, I. J. (2017). Habitat use and spatial fidelity of male South American sea lions during the nonbreeding period. Ecology and Evolution, 7(11), 3992–4002. 10.1002/ece3.2972 28616194 PMC5468127

[ece37337-bib-0012] Bhattacharyya, A. (1943). On a measure of divergence between two statistical populations defined by their probability distributions. Bulletin of the Calcutta Mathematical Society, 35, 99–109.

[ece37337-bib-0013] Bolnick, D. I. , Svanbäck, R. , Fordyce, J. A. , Yang, L. H. , Davis, J. M. , Hulsey, C. D. , & Forister, M. L. (2003). The ecology of individuals: Incidence and implications of individual specialization. The American Naturalist, 161(1), 1–28. 10.1086/343878 12650459

[ece37337-bib-0014] Boyd, I. L. , Arnould, J. P. , Barton, T. , & Croxall, J. P. (1994). Foraging behaviour of Antarctic fur seals during periods of contrasting prey abundance. Journal of Animal Ecology, 63(3), 703–713. 10.2307/5235

[ece37337-bib-0015] Burnham, K. , & Anderson, D. (2002). Model selection and multi‐model inference. 2, New York: Springer.

[ece37337-bib-0016] Calenge, C. (2006). The package *adehabitat* for the R software: A tool for the analysis of space and habitat use by animals. Ecological Modelling, 197, 516–519.

[ece37337-bib-0017] Camprasse, E. C. , Cherel, Y. , Arnould, J. P. Y. , Hoskins, A. J. , & Bost, C. A. (2017). Combined bio‐logging and stable isotopes reveal individual specialisations in a benthic coastal seabird, the Kerguelen shag. PLoS One, 12(3), e0172278. 28264057 10.1371/journal.pone.0172278 PMC5338780

[ece37337-bib-0018] Carroll, G. , Everett, J. D. , Harcourt, R. , Slip, D. , & Jonsen, I. (2016). High sea surface temperatures driven by a strengthening current reduce foraging success by penguins. Scientific Reports, 6(1), 22236. 10.1038/srep22236 26923901 PMC4770590

[ece37337-bib-0019] Charnov, E. L. (1976). Optimal foraging, the Marginal Value Theorem. Theoretical Population Biology, 9, 129–136. 10.1016/0040-5809(76)90040-X 1273796

[ece37337-bib-0020] Chilvers, B. L. (2008). Foraging site fidelity of lactating New Zealand sea lions. Journal of Zoology, 276(1), 28–36. 10.1111/j.1469-7998.2008.00463.x

[ece37337-bib-0021] Costa, D. P. (1993). The relationship between reproductive and foraging energetics and the evolution of the Pinnipedia. Symposia of the Zoological Society of London, 66, 293–314.

[ece37337-bib-0022] Costa, D. P. (2007). A conceptual model of the variation in parental attendance in response to environmental fluctuation: Foraging energetics of lactating sea lions and fur seals. Aquatic Conservation: Marine and Freshwater Ecosystems, 17, S44–S52.

[ece37337-bib-0023] Croxall, J. P. , Everson, I. , Kooyman, G. L. , Ricketts, C. , & Davis, R. W. (1985). Fur seal diving behaviour in relation to vertical distribution of krill. Journal of Animal Ecology, 54(1), 1–8. 10.2307/4616

[ece37337-bib-0024] Croxall, J. P. , Reid, K. , & Prince, P. A. (1999). Diet, provisioning and productivity responses of marine predators to differences in availability of Antarctic krill. Marine Ecology Progress Series, 177, 115–131. 10.3354/meps177115

[ece37337-bib-0025] Dall, S. R. , Bell, A. M. , Bolnick, D. I. , & Ratnieks, F. L. (2012). An evolutionary ecology of individual differences. Ecology Letters, 15(10), 1189–1198. 10.1111/j.1461-0248.2012.01846.x 22897772 PMC3962499

[ece37337-bib-0026] Dias, M. P. , Granadeiro, J. P. , Phillips, R. A. , Alonso, H. , & Catry, P. (2011). Breaking the routine: Individual Cory's shearwaters shift winter destinations between hemispheres and across ocean basins. Proceedings of the Royal Society B: Biological Sciences, 278(1713), 1786–1793. 10.1098/rspb.2010.2114 PMC3097831 21106591

[ece37337-bib-0027] Dingemanse, N. J. , & Dochtermann, N. A. (2013). Quantifying individual variation in behaviour: Mixed‐effect modelling approaches. Journal of Animal Ecology, 82(1), 39–54. 10.1111/1365-2656.12013 23171297

[ece37337-bib-0028] Doubleday, Z. A. , Clarke, S. M. , Li, X. , Pecl, G. T. , Ward, T. M. , Battaglene, S. , Frusher, S. , Gibbs, P. J. , Hobday, A. J. , Hutchinson, N. , & Hennings, S. M. (2013). Assessing the risk of climate change to aquaculture: A case study from south‐east Australia. Journal of Aquaculture Environment Interactions, 3(2), 163–175. 10.3354/aei00058

[ece37337-bib-0029] Estes, J. A. , Riedman, M. L. , Staedler, M. M. , Tinker, M. T. , & Lyon, B. E. (2003). Individual variation in prey selection by sea otters: Patterns, causes and implications. Journal of Animal Ecology, 72, 144–155.

[ece37337-bib-0030] Foster, S. A. (1999). The geography of behaviour: An evolutionary perspective. Trends in Ecology & Evolution, 14(5), 190–195. 10322532 10.1016/s0169-5347(98)01577-8

[ece37337-bib-0031] Frederiksen, M. , Edwards, M. , Richardson, A. J. , Halliday, N. C. , & Wanless, S. (2006). From plankton to top predators: Bottom‐up control of a marine food web across four trophic levels. Journal of Animal Ecology, 75(6), 1259–1268. 10.1111/j.1365-2656.2006.01148.x 17032358

[ece37337-bib-0032] Gibbs, C. F. , Tomczak, M. Jr , & Longmore, A. R. (1986). Nutrient regime of Bass Strait. Australian Journal of Marine & Freshwater Research, 37, 451–466.

[ece37337-bib-0033] Gutowsky, L. F. G. , Brownscombe, J. W. , Wilson, A. D. M. , Szekeres, P. , & Cooke, S. J. (2016). Improved performance, within‐individual consistency and between‐individual differences in the righting behaviour of the Caribbean sea star, *Oreaster reticulatus* . Behaviour, 153(13–14), 1763–1776.

[ece37337-bib-0034] Harris, S. M. , Raya Rey, A. , Zavalaga, C. , & Quintana, F. (2014). Strong temporal consistency in the individual foraging behaviour of Imperial Shags *Phalacrocorax atriceps* . Ibis, 156(3), 523–533.

[ece37337-bib-0035] Hatase, H. , Omuta, K. , & Tsukamoto, K. (2013). A mechanism that maintains alternative life histories in a loggerhead sea turtle population. Ecology, 94(11), 2583–2594. 24400510 10.1890/12-1588.1

[ece37337-bib-0036] Hijmans, R. J. (2020). raster: Geographic Data Analysis and Modeling. R package version 3.1‐5. https://cran.r‐project.org/package=raster

[ece37337-bib-0037] Hobday, A. J. , & Lough, J. M. (2011). Projected climate change in Australian marine and freshwater environments. Marine and Freshwater Research, 62(9), 1000–1014. 10.1071/MF10302

[ece37337-bib-0038] Hobday, A. J. , & Pecl, G. T. (2014). Identification of global marine hotspots: Sentinels for change and vanguards for adaptation action. Reviews in Fish Biology and Fisheries, 24(2), 415–425. 10.1007/s11160-013-9326-6

[ece37337-bib-0039] Hoskins, A. J. , & Arnould, J. P. Y. (2013). Temporal allocation of foraging effort in female Australian fur seals (*Arctocephalus pusillus doriferus*). PLoS One, 8(11), e79484. 10.1371/journal.pone.0079484 24244511 PMC3828376

[ece37337-bib-0040] Hoskins, A. J. , Costa, D. P. , Wheatley, K. E. , Gibbens, J. R. , & Arnould, J. P. Y. (2015). Influence of intrinsic variation on foraging behaviour of adult female Australian fur seals. Marine Ecology Progress Series, 526, 227–239. 10.3354/meps11200

[ece37337-bib-0041] Jeanniard‐du‐Dot, T. , Trites, A. W. , Arnould, J. P. Y. , & Guinet, C. (2017). Reproductive success is energetically linked to foraging efficiency in Antarctic fur seals. PLoS One, 12(4), e0174001. 28453563 10.1371/journal.pone.0174001 PMC5409505

[ece37337-bib-0042] Jensen, A. J. , Finstad, B. , Fiske, P. , Diserud, O. H. , & Thorstad, E. B. (2020). Repeatable individual variation in migration timing in two anadromous salmonids and ecological consequences. Ecology and Evolution, 10, 11727–11738. 33144996 10.1002/ece3.6808 PMC7593174

[ece37337-bib-0043] Kernaléguen, L. , Cherel, Y. , Knox, T. C. , Baylis, A. M. M. , & Arnould, J. P. Y. (2015). Sexual niche segregation and gender‐specific individual specialisation in a highly dimorphic marine mammal. PLoS One, 10(8), 1–15. 10.1371/journal.pone.0133018 PMC4526469 26244371

[ece37337-bib-0044] Kernaléguen, L. , Dorville, N. , Ierodiaconou, D. , Hoskins, A. J. , Baylis, A. M. , Hindell, M. A. , Semmens, J. , Abernathy, K. , Marshall, G. J. , Cherel, Y. , & Arnould, J. P. Y. (2016). From video recordings to whisker stable isotopes: A critical evaluation of timescale in assessing individual foraging specialisation in Australian fur seals. Oecologia, 180(3), 657–670. 10.1007/s00442-015-3407-2 26233674

[ece37337-bib-0045] Kirkman, S. P. , Costa, D. P. , Harrison, A. L. , Kotze, P. G. H. , Oosthuizen, W. H. , Weise, M. J. , Botha, J. A. , & Arnould, J. P. Y. (2019). Dive behaviour and foraging effort of female Cape fur seals *Arctocephalus pusillus pusillus* . Royal Society Open Science, 6(10), 191369. 10.1098/rsos.191369 31824733 PMC6837185

[ece37337-bib-0046] Kirkwood, R. , & Arnould, J. P. Y. (2011). Foraging trip strategies and habitat use during late pup rearing by lactating Australian fur seals. Australian Journal of Zoology, 59(4), 216–226. 10.1071/ZO11080

[ece37337-bib-0047] Kirkwood, R. , Pemberton, D. , Gales, R. , Hoskins, A. J. , Mitchell, T. , Shaughnessy, P. D. , & Arnould, J. P. Y. (2010). Continued population recovery by Australian fur seals. Marine and Freshwater Research, 61(6), 695–701.

[ece37337-bib-0048] Knox, T. C. , Baylis, A. M. M. , & Arnould, J. P. Y. (2017). Habitat use and diving behaviour of male Australian fur seals. Marine Ecology Progress Series, 566, 243–256. 10.3354/meps12027

[ece37337-bib-0049] Knox, T. C. , Baylis, A. M. M. , & Arnould, J. P. Y. (2018). Foraging site fidelity in male Australian fur seals. Marine Biology, 165(7), 1–12. 10.1007/s00227-018-3368-1

[ece37337-bib-0050] Kuhn, C. E. , Ream, R. R. , Sterling, J. T. , Thomason, J. R. , & Towell, R. G. (2014). Spatial segregation and the influence of habitat on the foraging behavior of northern fur seals (*Callorhinus ursinus*). Canadian Journal of Zoology, 92(10), 861–873.

[ece37337-bib-0051] Last, P. R. , White, W. T. , Gledhill, D. C. , Hobday, A. J. , Brown, R. , Edgar, G. J. & Pecl, G. (2011). Long‐term shifts in abundance and distribution of a temperate fish fauna: A response to climate change and fishing practices. Global Ecology and Biogeography, 20(1), 58–72. 10.1111/j.1466-8238.2010.00575.x

[ece37337-bib-0052] Lowther, A. D. , Harcourt, R. G. , Goldsworthy, S. D. , & Stow, A. (2012). Population structure of adult female Australian sea lions is driven by fine‐scale foraging site fidelity. Animal Behaviour., 83(3), 691–701. 10.1016/j.anbehav.2011.12.015

[ece37337-bib-0053] Luque, S. (2019) diveMove. R package version 1.4.5.

[ece37337-bib-0054] Mattern, T. , Ellenberg, U. , Houston, D. M. , & Davis, L. S. (2007). Consistent foraging routes and benthic foraging behaviour in yellow‐eyed penguins. Marine Ecology Progress Series, 343, 295–306. 10.3354/meps06954

[ece37337-bib-0055] McHuron, E. A. , Hazen, E. , & Costa, D. P. (2018). Constrained by consistency? Repeatability of foraging behavior at multiple timescales for a generalist marine predator. Marine Biology, 165(8), 1–13. 10.1007/s00227-018-3382-3

[ece37337-bib-0056] Meyers, N. (2019). The cost of a meal: Foraging ecology of female Australian fur seals. Australia: Deakin University.

[ece37337-bib-0058] Nakagawa, S. , & Schielzeth, H. (2010). Repeatability for Gaussian and non‐Gaussian data: A practical guide for biologists. Biological Reviews, 85(4), 935–956. 10.1111/j.1469-185X.2010.00141.x 20569253

[ece37337-bib-0059] Patrick, S. C. , & Weimerskirch, H. (2017). Reproductive success is driven by local site fidelity despite stronger specialisation by individuals for large‐scale habitat preference. Journal of Animal Ecology, 86(3), 674–682. 10.1111/1365-2656.12636 28117897

[ece37337-bib-0060] Pinheiro, J. , Bates, D. , DebRoy, S. , Sarkar, D. , & Team RC . (2019) nlme: Linear and Nonlinear Mixed Effects Models. R package version 3.1‐140. https://cran.r‐project.org/package=nlme

[ece37337-bib-0061] Speakman, C. N. , Hoskins, A. J. , Hindell, M. A. , Costa, D. , Hartog, J. R. , Hobday, A. J. , & Arnould, J. P. Y. (2020). Environmental influences on foraging effort, success and efficiency in female Australian fur seals. Scientific Reports, 10, 17710–17710. 10.1038/s41598-020-73579-y 33077806 PMC7572486

[ece37337-bib-0062] Stoffel, M. A. , Nakagawa, S. , & Schielzeth, H. (2017). rptR: Repeatability estimation and variance decomposition by generalized linear mixed‐effects models. Methods in Ecology and Evolution, 8(11), 1639–1644. 10.1111/2041-210X.12797

[ece37337-bib-0063] Sutton, G. , Pichegru, L. , Botha, J. A. , Kouzani, A. Z. , Adams, S. , Bost, C. A. & Arnould, John P. Y. (2020). Multi‐predator assemblages, dive type, bathymetry and sex influence foraging success and efficiency in African penguins. PeerJ, 8, e9380. 10.7717/peerj.9380 32655991 PMC7333648

[ece37337-bib-0064] Switzer, P. V. (1993). Site fidelity in predictable and unpredictable habitats. Evolutionary Ecology, 7(6), 533–555. 10.1007/BF01237820

[ece37337-bib-0065] R Core Development Team (2019). R: A language and environment for statistical computing. R Foundation for Statistical Computing. https://www.r‐project.org/

[ece37337-bib-0066] Tinker, M. T. , Bentall, G. , & Estes, J. A. (2008). Food limitation leads to behavioral diversification and dietary specialization in sea otters. Proceedings of the National Academy of Sciences USA, 105(2), 560–565. 10.1073/pnas.0709263105 PMC2206575 18195370

[ece37337-bib-0067] Visconti, V. , Trip, E. D. L. , Griffiths, M. H. , & Clements, K. D. (2018). Life‐history traits of the leatherjacket *Meuschenia scaber*, a long‐lived monacanthid. Journal of Fish Biology, 92(2), 470–486. 29431226 10.1111/jfb.13529

[ece37337-bib-0068] Volpov, B. L. , Rosen, D. A. , Hoskins, A. J. , Lourie, H. J. , Dorville, N. , Baylis, A. M. , Wheatley, K. E. , Marshall, G. , Abernathy, K. , Semmens, J. , Hindell, M. A. , & Arnould, J. P. Y. (2016). Dive characteristics can predict foraging success in Australian fur seals (*Arctocephalus pusillus doriferus*) as validated by animal‐borne video. Biology Open, 5(3), 262–271. 26873950 10.1242/bio.016659 PMC4810750

[ece37337-bib-0069] Williams, H. , & Pullen, G. (1993). Schooling behaviour of jack mackerel, *Trachurus declivis* (Jenyns), observed in the Tasmanian purse seine fishery. Marine and Freshwater Research, 44(4), 577–587. 10.1071/MF9930577

[ece37337-bib-0070] Woo, K. J. , Elliott, K. H. , Davidson, M. , Gaston, A. J. , & Davoren, G. K. (2008). Individual specialization in diet by a generalist marine predator reflects specialization in foraging behaviour. Journal of Animal Ecology, 77(6), 1082–1091. 10.1111/j.1365-2656.2008.01429.x 18624834

[ece37337-bib-0071] Ydenberg, R. C. , Welham, C. V. J. , Schmid‐Hempel, R. , Schmid‐Hempel, P. , & Beauchamp, G. (1994). Time and energy constraints and the relationships between currencies in foraging theory. Behavioral Ecology, 5(1), 28–34.

[ece37337-bib-0072] Zuur, A. , Ieno, E. N. , & Elphick, C. S. (2010). A protocol for data exploration to avoid common statistical problems. Methods in Ecology and Evolution, 1(1), 3–14.

[ece37337-bib-0073] Zuur, A. , Ieno, E. N. , Walker, N. , Saveliev, A. A. , & Smith, G. M. (2009). Mixed effects models and extensions in ecology with R. New York: Springer. 10.1007/978-0-387-87458-6

