# Supplementary material for: Intertrip consistency in hunting behavior improves foraging success and efficiency in a marine top predator
Source: Ecol Evol. 2021 Mar 13;11(9):4428–41. doi: 10.1002/ece3.7337 (PMC8093728; doi:10.1002/ece3.7337)
Supplement: Supplementary file 1 — Supplementary Material [file ECE3-11-4428-s001.pdf]

Inter-trip consistency in hunting behaviour improves foraging success and efficiency in a marine top predator

Short title: Behavioural consistency in female AUFS

Cassie N. Speakman[1], Sebastian T. Lloyd[1], Elodie C. M. Camprasse[1], Andrew J. Hoskins[2], Mark A. Hindell[3], Daniel P. Costa[4] and John P. Y. Arnould[1]

[1]Deakin University, School of Life and Environmental Sciences, Burwood, Victoria, Australia

[2]CSIRO Health and Biosecurity, Townsville, Qld, Australia

[3]Institute for Marine and Antarctic Studies, University of Tasmania, Hobart, Tasmania, Australia

[4]Ecology and Evolutionary Biology Department, University of California Santa Cruz, Santa Cruz, California, USA

*Corresponding author – cspeakman@deakin.edu.au

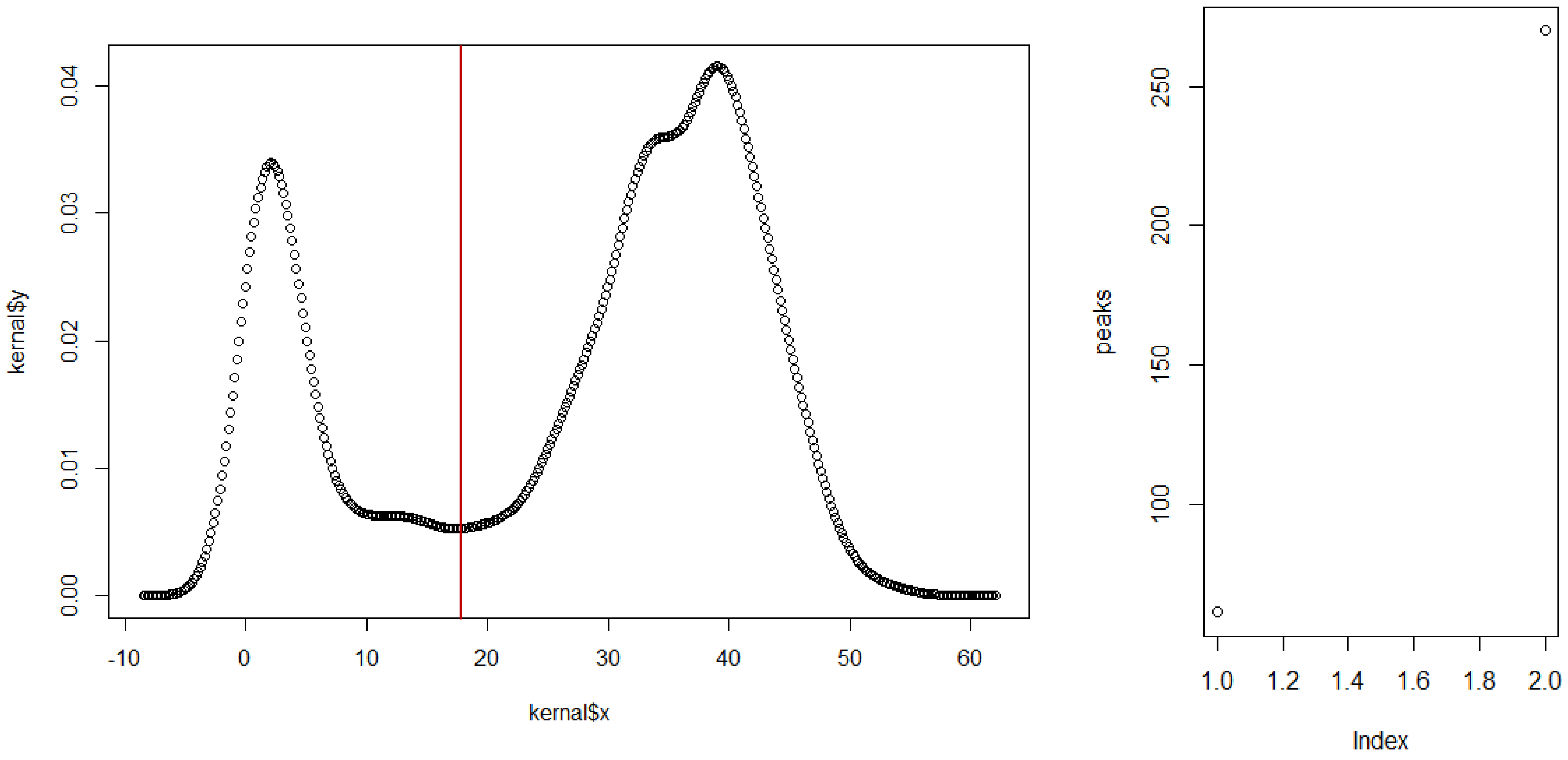

Figure S1. Example of classification of dive types using the bimodal distribution of the kernel density estimate dives. Points falling to the right of the nadir (red line) are classified as benthic, whereas dives to the left of the nadir are classified as pelagic. The quantity of each dive type in this example is 61 and 270 for pelagic and benthic dives, respectively.

Table S1. Summary of at-sea movements and dive behaviour for female Australian fur seals from Kanowna Island, Bass Strait, south-eastern Australia, 2006-2019. Data are presented as Means ± SE (SD for bearing). Means are derived from the means ≥3 foraging trips per individual. Bearing is calculated such that a bearing of 0º indicates direct northward movement from the colony and 180º indicated direct southward movement from the colony.

ID	Year	No. trips	Bearing(º)	Maximum distance from colony (km)	Total distance travelled (km)	Trip duration (h)	Modal dive depth (m)	Dive duration (s)	Dive rate ($m \cdot h^{-1}$)	Daytime dives (%)	Benthic dives (%)	Foraging Site Fidelity Index	Foraging Trip Success Index	Foraging Trip Efficiency Index
1	2006	5	203.4 ± 82.6	124.2 ± 55.6	621.3 ± 277.9	152.4 ± 68.2	81.7 ± 36.5	174.1 ± 77.9	713.0 ± 318.9	23.8 ± 10.6	89.3 ± 3.1	0.12	12.48 ± 0.77	0.19 ± 0.03
2	2007	9	187.0 ± 119.6	51.0 ± 17.0	298.1 ± 99.4	70.9 ± 23.6	76.6 ± 25.5	169.2 ± 56.4	2315.5 ± 771.8	27.2 ± 9.1	75.1 ± 4.8	0.07	9.92 ± 0.45	0.06 ± 0.01
3	2007	10	233.9 ± 43.4	42.6 ± 13.5	268.4 ± 84.9	73.4 ± 23.2	73.4 ± 23.2	183.1 ± 57.9	680.8 ± 215.3	47.6 ± 15.1	63.7 ± 7.0	0.35	6.52 ± 0.70	0.07 ± 0.01
4	2008	3	257.8 ± 88.2	87.8 ± 50.7	312.2 ± 180.2	95.0 ± 54.9	73.7 ± 42.6	228.8 ± 132.1	785.1 ± 453.3	48.9 ± 28.3	90.3 ± 6.9	0.03	6.12 ± 1.36	0.14 ± 0.03
5	2008	11	172.5 ± 78.6	42.5 ± 12.8	203.4 ± 61.3	63.2 ± 19.0	74.3 ± 22.4	196.1 ± 59.1	1153.3 ± 347.7	78.7 ± 23.7	90.2 ± 0.9	0.21	11.76 ± 0.54	0.05 ± 0.01
6	2008	3	161.0 ± 56.3	118.4 ± 68.3	322.7 ± 186.3	71.0 ± 41.0	82.6 ± 47.7	237.0 ± 136.8	1973.7 ± 1139.5	49.2 ± 28.4	91.5 ± 1.7	0.05	8.85 ± 0.57	0.14 ± 0.02
7	2009	4	248.8 ± 35.2	78.5 ± 39.3	684.5 ± 342.3	220.2 ± 110.1	80.8 ± 40.4	153.5 ± 76.8	867.9 ± 433.9	29.5 ± 14.7	84.5 ± 4.4	0.64	17.88 ± 0.44	0.22 ± 0.03
8	2009	3	180.8 ± 76.9	154.2 ± 89.1	571.6 ± 330.0	121.8 ± 70.3	81.5 ± 47.0	184.2 ± 106.3	1378.1 ± 795.6	43.3 ± 25.0	73.4 ± 7.4	0.09	10.72 ± 0.71	0.21 ± 0.03
9	2019	6	140.6 ± 140.4	123.8 ± 50.5	836.1 ± 341.3	174.2 ± 71.1	71.0 ± 29.0	177.8 ± 72.6	1754.3 ± 716.2	45.0 ± 18.4	76.9 ± 5.7	0.18	8.97 ± 0.59	0.09 ± 0.02
10	2011	5	185.2 ± 56.2	103.7 ± 46.4	516.9 ± 231.2	152.2 ± 68.1	84.1 ± 37.6	200.6 ± 89.7	701.0 ± 313.5	59.2 ± 26.5	91.6 ± 5.2	0.14	11.60 ± 0.59	0.25 ± 0.04
11	2012	5	207.0 ± 28.1	82.8 ± 37.0	341.0 ± 152.5	107.4 ± 48.0	83.8 ± 37.5	190.9 ± 85.4	636.9 ± 284.8	53.1 ± 23.7	96.3 ± 0.9	0.60	12.83 ± 0.34	0.19 ± 0.01
12	2012	4	167.2 ± 97.5	68.2 ± 34.1	399.3 ± 199.7	110.7 ± 55.3	84.1 ± 42.0	194.0 ± 97.0	838.2 ± 419.1	35.1 ± 17.5	92.2 ± 3.3	0.11	12.24 ± 0.38	0.20 ± 0.03
13	2013	4	147.9 ± 39.1	124.9 ± 62.4	216.3 ± 108.2	73.7 ± 36.9	7.6 ± 3.8	81.1 ± 40.6	1154.4 ± 577.2	17.0 ± 8.5	56.7 ± 4.9	0.12	13.06 ± 0.63	0.08 ± 0.01
14	2013	7	208.0 ± 101.5	99.4 ± 37.6	395.1 ± 149.3	99.8 ± 37.7	61.5 ± 23.3	118.7 ± 44.8	642.0 ± 242.7	37.5 ± 14.2	65.3 ± 6.1	0.30	14.59 ± 0.52	0.17 ± 0.02
15	2013	12	233.9 ± 121.8	31.0 ± 8.9	150.3 ± 43.4	57.4 ± 16.6	72.3 ± 20.9	147.7 ± 42.6	680.4 ± 196.4	39.5 ± 11.4	86.8 ± 3.3	0.25	15.93 ± 0.70	0.08 ± 0.01
16	2013	3	117.6 ± 34.9	138.1 ± 79.7	279.9 ± 161.6	74.2 ± 42.9	33.6 ± 19.4	96.0 ± 55.4	1556.9 ± 898.9	52.4 ± 30.3	51.8 ± 5.8	0.16	10.07 ± 0.44	0.07 ± 0.01
17	2013	8	164.2 ± 70.9	49.6 ± 17.5	264.5 ± 93.5	94.2 ± 33.3	80.0 ± 28.3	229.3 ± 81.1	953.1 ± 337.0	63.1 ± 22.3	96.7 ± 0.9	0.31	11.12 ± 0.37	0.19 ± 0.03
18	2014	3	167.4 ± 83.7	208.2 ± 120.2	346.7 ± 200.2	64.0 ± 36.9	8.2 ± 4.7	67.9 ± 39.2	1850.0 ± 1068.1	19.0 ± 10.9	31.9 ± 6.9	0.33	9.38 ± 0.52	0.08 ± 0.01
19	2014	10	207.0 ± 77.4	76.4 ± 24.2	322.3 ± 101.9	82.3 ± 26.0	82.7 ± 26.1	201.5 ± 63.7	457.8 ± 144.8	41.3 ± 13.0	82.8 ± 2.8	0.58	10.94 ± 0.38	0.15 ± 0.01
20	2014	6	201.8 ± 68.0	92.7 ± 37.9	341.3 ± 139.4	88.7 ± 36.2	58.5 ± 23.9	136.1 ± 55.5	584.9 ± 238.8	13.5 ± 5.5	67.7 ± 5.1	0.15	11.03 ± 0.55	0.12 ± 0.01
21	2014	6	181.3 ± 74.2	71.3 ± 29.1	432.7 ± 176.7	125.1 ± 51.1	84.7 ± 34.6	182.6 ± 74.6	514.6 ± 210.1	53.6 ± 21.9	90.5 ± 2.9	0.16	13.57 ± 0.42	0.19 ± 0.01
22	2016	3	175.6 ± 85.0	132.7 ± 76.6	628.9 ± 363.1	148.0 ± 85.5	6.6 ± 3.8	59.8 ± 34.5	1147.5 ± 662.5	20.9 ± 12.0	26.0 ± 6.8	0.01	6.05 ± 0.31	0.07 ± 0.02
23	2016	5	188.9 ± 94.9	97.1 ± 43.4	494.3 ± 221.1	137.5 ± 61.5	37.4 ± 16.7	124.9 ± 55.9	652.9 ± 292.0	21.2 ± 9.5	52.8 ± 12.2	0.17	6.68 ± 0.21	0.13 ± 0.02
24	2017	3	190.9 ± 31.2	36.3 ± 20.9	310.9 ± 179.5	89.2 ± 51.5	31.3 ± 18.1	59.5 ± 34.4	276.5 ± 159.7	12.5 ± 7.2	68.2 ± 11.3	0.02	9.82 ± 0.83	0.11 ± 0.01

25	2017	4	211.8 ± 121.5	68.4 ± 34.2	290.3 ± 145.2	97.5 ± 48.7	26.2 ± 13.1	112.8 ± 56.4	420.4 ± 210.2	37.1 ± 18.5	40.8 ± 5.3	0.25	9.55 ± 0.77	0.08 ± 0.01
26	2017	5	200.4 ± 135.1	83.5 ± 37.3	334.7 ± 149.7	99.4 ± 44.5	5.8 ± 2.6	39.7 ± 17.7	412.9 ± 184.6	16.4 ± 7.4	30.0 ± 14.7	0.21	7.44 ± 0.51	0.05 ± 0.02
27	2017	6	171.5 ± 12.1	154.4 ± 63.0	424.4 ± 173.2	85.7 ± 35.0	45.2 ± 18.5	137.6 ± 56.2	4171.7 ± 1703.1	26.5 ± 10.8	50.7 ± 8.0	0.25	10.31 ± 0.71	0.12 ± 0.03
28	2018	5	50.8 ± 49.2	96.7 ± 43.3	229.1 ± 102.4	59.4 ± 26.6	30.0 ± 13.4	167.7 ± 75.0	2476.1 ± 1107.4	84.5 ± 37.8	94.0 ± 1.3	0.60	7.53 ± 0.40	0.12 ± 0.03
29	2018	6	207.3 ± 73.7	108.7 ± 44.4	393.9 ± 160.8	95.3 ± 38.9	82.1 ± 33.5	183.9 ± 75.1	876.4 ± 357.8	56.5 ± 23.1	91.1 ± 4.2	0.20	12.47 ± 0.27	0.17 ± 0.01
30	2019	19	237.2 ± 118.8	100.6 ± 23.1	473.1 ± 108.5	119.1 ± 27.3	37.5 ± 8.6	120.0 ± 27.5	747.2 ± 171.4	35.8 ± 8.2	50.6 ± 6.0	0.17	8.92 ± 0.21	0.14 ± 0.01
31	2019	12	265.1 ± 115.4	61.4 ± 17.7	433.1 ± 125.0	107.1 ± 30.9	73.7 ± 21.3	163.9 ± 47.3	1310.2 ± 378.2	58.8 ± 17.0	71.9 ± 2.9	0.44	12.73 ± 0.90	0.14 ± 0.02
32	2019	10	200.3 ± 86.8	91.8 ± 29.0	520.7 ± 164.7	124.0 ± 39.2	15.0 ± 4.7	70.0 ± 22.1	1181.8 ± 373.7	22.5 ± 7.1	62.9 ± 10.6	0.08	12.55 ± 0.31	0.10 ± 0.02
33	2019	15	248.8 ± 164.0	49.3 ± 12.7	288.3 ± 74.4	65.0 ± 16.8	75.0 ± 19.4	151.5 ± 39.1	1688.8 ± 436.0	54.0 ± 13.9	93.6 ± 2.6	0.24	17.18 ± 1.11	0.13 ± 0.00
34	2019	16	243.8 ± 80.7	63.2 ± 15.8	405.3 ± 101.3	116.1 ± 29.0	80.6 ± 20.2	187.6 ± 46.9	979.5 ± 244.9	69.4 ± 17.3	93.4 ± 3.1	0.08	13.94 ± 0.21	0.17 ± 0.01
	**Mean**		203.5 ± 49.1	81.1 ± 3.3	390.2 ± 13.4	103.5 ± 3.7	62.2 ± 2.0	155.3 ± 3.6	1117.3 ± 105.5	44.1 ± 1.7	75.6 ± 1.7	0.2 ± 0.0	11.02 ± 0.51	0.13 ± 0.01
